# Long-Term Trends for Blue Mussels from the German Environmental Specimen Bank Show First Evidence of Munition Contaminants Uptake

**DOI:** 10.3390/toxics11040347

**Published:** 2023-04-07

**Authors:** Jennifer Susanne Strehse, Tobias Hartwig Bünning, Jan Koschorreck, Anita Künitzer, Edmund Maser

**Affiliations:** 1 Institute of Toxicology and Pharmacology for Natural Scientists, University Medical School Schleswig-Holstein, Brunswiker Straße 10, 24105 Kiel, Germanymaser@toxi.uni-kiel.de (E.M.); 2 German Environment Agency, Wörlitzer Platz 1, 06844 Dessau, Germany

**Keywords:** energetic compounds, trinitrotoluene, explosives, blue mussels, munitions, environmental specimen bank

## Abstract

Submerged munitions are present in marine waters across the globe. They contain energetic compounds (ECs), such as TNT and metabolites thereof, which are considered carcinogenic, exhibit toxic effects in marine organisms, and may affect human health. The aim of this study was to investigate the occurrence of ECs and their trends in blue mussels from the annual collections of the German Environmental Specimen Bank sampled over the last 30 years at three different locations along the coastline of the Baltic and North Sea. Samples were analyzed by GC-MS/MS for 1,3-dinitrobenzene (1,3-DNB), 2,4-dinitrotoluene (2,4-DNT), 2,4,6-trinitrotoluene (TNT), 2-amino-4,6-dinitrotoluene (2-ADNT), and 4-amino-2,6-dinitrotoluene (4-ADNT). The first signals indicating trace levels of 1,3-DNB were observed in samples from 1999 and 2000. ECs were also found below the limit of detection (LoD) in subsequent years. From 2012 onwards, signals just above the LoD were detected. The highest signal intensities of 2-ADNT and 4-ADNT, just below the LoQ (0.14 ng/g d.w. and 0.17 ng/g d.w., respectively), were measured in 2019 and 2020. This study clearly shows that corroding submerged munitions are gradually releasing ECs into the waters that can be detected in randomly sampled blue mussels, even though the concentrations measured are still in the non-quantifiable trace range.

## 1. Introduction

Discarded munition materials and unexploded ordnances (UXOs) are present in marine waters across the world [1,2], the majority of which resulted from the First and Second World Wars. These munition relics entered marine waters and coastal areas during wartime operations and include fired but unexploded ammunitions or cargo from sunken vessels and downed fighter aircraft [3,4,5]. After the World Wars, military munitions entered the world’s oceans through the disposal of unwanted munition materials in attempts to demilitarize defeated nations [4,6]. Unlike other ubiquitous contaminants, such as waste oils, microplastics, and legacy pollutants, discarded military munitions and UXOs are primarily hotspot problems. Enormous quantities of UXOs and dumped munition materials can be present in these hotspots, whereas other parts of the world’s oceans may not be polluted. For instance, it is estimated that, during the Second World War, over 7800 ships and vessels sunk worldwide, many of them loaded with ammunition or other kinds of military munitions [7,8]. Furthermore, hotspots were “created” by the dumping of relic munition materials. This lead, for instance, to a quantity of at least 1.6 million metric tons of conventional munitions being dumped in the coastal areas of the German North and Baltic Sea [4].

A number of studies in recent years have shown that the metal casings of explosive remnants of war exposed to seawater corrode over time (e.g., [9,10,11]) and release toxic energetic compounds into the surrounding water body. The corrosion rate of dumped munitions is difficult to estimate and dependent on environmental factors, such as salinity, water temperature, and current. One study on Second World War-era shipwrecks in Micronesia measured corrosion rates in the order of 0.1 mm y^−1^ [12], suggesting that corrosion depths after 70 years may be nearly 1 cm.

Most of the UXOs and discarded munition materials contain 2,4,6-trinitrotoluene (TNT) as the main energetic compound [13]. In addition to its explosive properties, TNT, which remains present in the environment decades after the dumping of munitions, poses various hazards to human health and the marine ecosystem.

Several studies have shown that TNT and its amino transformation metabolites, such as 2-amino-4,6-dinitrotoluene (2-ADNT) and 4-amino-2,6-dinitrotoluene (4-ADNT), have acute and chronic toxic effects on a wide range of marine organisms. These include, for instance, the impairment of germination and germling length and reductions in algae cell numbers [14]; effects on the survival of redfish larvae [14]; effects on the embryo-larval development and survival of the blue mussel *M. galloprovincialis* [15]; effects on the survival of fathead minnow, channel fish, and rainbow trout [16]; DNA damage in zebrafish embryos [17]; and oxidative stress in blue mussels (*Mytilus* spp.) [18,19]. In humans, TNT and its metabolites are mutagenic and of relevant urothelial carcinogenicity, as well as toxic to the liver, kidneys, eyes, skin, blood system, and spleen [20]. The mechanisms underlying the toxicity and carcinogenicity of TNT and its metabolites are based on the induction of oxidative stress and reactive oxygen species (ROS) in biological tissues [20]. Another player in TNT mutagenicity could be the 4-hydroxylamino-2,6-dinitrotoluene (4-HADNT) intermediate, which is a product of cytochrome P450 catalysis on the way to 4-ADNT [21]. 4-HADNT has been shown to cause severe DNA damage to the male reproductive system in rats [22].

TNT, its metabolites, and other common energetic compounds have been detected in water, sediment, and biota adjacent to sea-dumped munitions [23,24,25,26] and in water and sediment samples from a former military shooting range [27]. Recently, a biomonitoring study with blue mussels (*Mytilus* spp.) showed that TNT leaks from moored mines and can be detected in the tissue of exposed mussels [28]. These mussels were exposed to corroding moored mines for a relatively short period of just three months [28]. This provided evidence that TNT from marine World War relicts enters the food chain [18]. Since then, the carcinogenicity and mutagenicity of TNT and its metabolites have been the subject of growing public and scientific debate, as the consumption of contaminated fish and other seafood by human consumers cannot be ruled out [23,29,30].

The development of monitoring and detection methods is of high relevance for the enhancement of the localization and mapping of munitions on the seafloor, especially since the exact locations of large amounts of munitions remain unknown. A continuous biomonitoring program has been carried out for the last two decades by the Netherlands’ Organization for Applied Scientific Research (TNO) in dumping sites in the Netherlands in the Eastern Scheldt near Zierikzee and in the Wadden Sea (North Sea) [11]. In addition to active biomonitoring using mussels (*Mytilus edulis*), sediment and water samples are routinely analyzed to infer deviations in the marine species composition or possible risks to human health due to contaminations with energetic compounds. [31]. Other methods of monitoring include the application of passive sampling systems, and special polyethersulfone membranes (polar organic chemical integrative sampler (POCIS)) have been used to monitor ECs in Swiss fresh water lakes [32] and marine environments [33,34]. Interestingly, by comparing the effectiveness of passive sampling systems with grab sampling results, Lotufo et al. [35] successfully demonstrated the accuracy of POCIS in estimating concentrations of energetic compounds, thus underlining that POCIS provide reliable temporal integration of changing environmental concentrations for common energetic compounds.

Upon performing a biomonitoring approach, up to 60-fold higher concentrations of energetic compounds were recorded in blue mussels (*Mytilus* spp.) that were present for three months in the vicinity of chunks of hexanite—which are composed of approximately 60–70% TNT—compared to those mussels that were exposed close to corroding but reasonably intact mines [18,28,29]. The main sources of exposed chunks of hexanite in the marine environment are dumped excess munitions from the Second World War and, to a larger extent, blast-in-place operations (BiPs) used to defuse dangerous unexploded ordnance; for example in shipping channels or in areas designated for construction of offshore wind parks [29]. The possibility of complete corrosion of the metal casings is also a major problem for the detection and associated localization of munitions in the sea. Without the metal casings, it is not possible to detect UXOs and other dumped munition materials using magnetometry-based methods.

For more than 40 years, mussels, such as blue mussels, have been used as bioindicators in contaminant monitoring [36] because of their sedentary nature, worldwide distribution, easy accessibility, and resistance to certain pollutants. Natural substances and anthropogenic chemicals dissolved in seawater or bound to particles are taken up by mussels at a filtration rate of about 2–3 L of seawater per hour and can be measured in the mussel soft body [36]. Recently, blue mussels have successfully been tested as indicators to monitor the leakage of explosive chemicals from munitions into the marine environment [37]. Since the 1980s, the German Environmental Specimen Bank (ESB) has systematically collected, analyzed, and archived blue mussels and 14 other species from the marine, freshwater, and terrestrial compartments. The ESB is part of the German long-term monitoring infrastructure in support of environmental policy and regulation [38]. The samples can be used at any time to examine temporal trends in environmental stressors that were not known to be problematic at the time of sampling, could not be analytically detected, or were ignored. Due to the storage of the samples at temperatures below −130 °C, the degradation and transformation processes within the tissues are stopped and the actual concentrations at the time of sampling can be analyzed retrospectively. Recent examples of retrospective analyses include metals [39]; legacy and emerging chlorinated, brominated, and fluorinated contaminants; and microplastics [40,41,42].

The aim of our study was to investigate the occurrence of and trends for explosive chemicals and their metabolites in blue mussels from the annual collections of the German Environmental Specimen Bank sampled in the North Sea and Baltic Sea over the last 35 years. This made it possible to not only identify possible hazards to human health and ecosystems but also draw conclusions about the condition of the munitions lying in the seabed at the sampling regions.

## 2. Materials and Methods

### 2.1. Sample Locations

**Figure 1:**
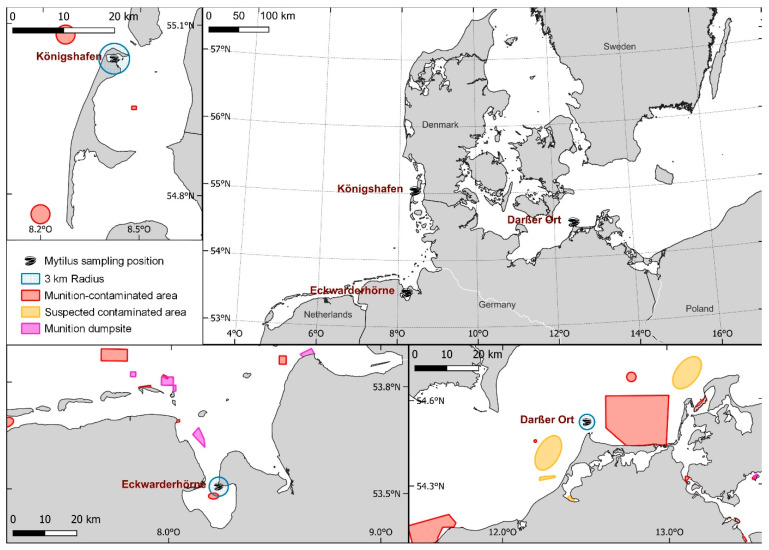
Map of the *Mytilus* sampling areas of the Environmental Specimen Bank: “Königshafen” on the island of Sylt (N55°2.3, E8°25.5), “Eckwarderhörne” (N53°31.2, E8°13.8) in the German North Sea, and “Darßer Ort” (N54°29.7, E12°31.6) in the Baltic. The officially known dumpsites, munition-contaminated areas, and suspected areas are shown as colored spaces (data provided by amucad.org). A 3 km radius was drawn around the sampling positions to show their proximity to possible munition deposits.

Blue mussels were sampled at three different locations—one at the coastline of the Baltic Sea and two in the North Sea region. The mussels analyzed in this study had been collected since 1985 in the North Sea region of Eckwarderhörne, located in the Lower Saxony Wadden Sea; since 1986 close to the island of Sylt (Königshafen), located in the Schleswig-Holstein Wadden Sea (North Sea); and since 1992 at Darßer Ort in the Bodden National Park of Western Pomerania (Baltic Sea) (Figure 1). According to the Ammunition Cadastre Sea (AmuCad https://amucad.org/ accessed on 5 November 2022), dumped munitions and UXOs from both war and post-war activities can be assumed to exist in all three sampling regions, including in designated dumping areas. Some areas have not been thoroughly investigated, so the amount of munitions and the number of contaminated sites may be larger than previously anticipated.

The sampling region Eckwarderhörne is contaminated with various types of munitions. Estimates suggest that 650,000 to 1.2 million tons of conventional munitions, including heavy shells and depth charges, were dumped close to the sampling area at the munition dumpsite Jade Hooksielplate (Figure 1) [4]. Another source estimates at least 900,000 tons of discarded munition materials within this area [43]. The munition-contaminated area Innere Jappensand/Jade, which lies partly in the 3 km radius around the sampling position (Figure 1), is littered with an unknown amount of different kinds of munitions; e.g., depth charges [4]. However, a fully comprehensive evaluation of historical data in this area is still pending.

The island of Sylt (Figure 1) was used for military purposes during the Second World War (harbors and Westerland airport) and many of the remaining munitions (mainly light ammunition, such as 15 cm grenades) were dumped in the sea—e.g., between the island and the German mainland—from June to August 1945 [4]. However, the exact dumping sites and quantities are unknown and still the subject of ongoing historical research.

According to Böttcher et al. [4], there are several known and suspected areas contaminated with munitions in the Baltic Sea sampling area Darßer Ort (Figure 1). Among others, this coastal area was the site of an anti-aircraft training range. Similar to the other two sampling sites, the presence of all types of munitions is therefore expected. Dumping of munitions in this region can also not be ruled out. A final investigation of the actual contamination with regard to the type and quantity of military munitions present and the spatial distribution of contamination has not yet been completed. Further evaluation of historical documents in this area is also still pending.

### 2.2. Sampling and Sample Processing for Blue Mussels (Mytilus edulis Complex)

Sample collection and processing is highly standardized and follows standard ESB protocols [44]. Mussels are collected every two months at two areas in the North Sea and twice a year (June and November/December) at one area in the Baltic Sea in selected sampling sites that have sufficiently large stocks and are stable over the long term. To reduce natural variability, certain length classes are sampled that are typical for at least two-year-old individuals. These length classes have been determined for each sampling site using screening methods and have been reviewed regularly over the past decades. A subsample of 50 mussels is biometrically characterized (e.g., shell length, shell weight, and soft body weight). The remaining minimum 50 mussels are used for initial chemical characterization and long-term storage. The cold chain begins immediately after sampling in the field, when the mussels are frozen at −130 °C over liquid nitrogen and transported to the laboratories in this manner. There, after a short thawing period, the shells are removed under a clean room bench, the frozen mussel soft bodies are cryo-milled to a homogeneous powder [45], and annual composite samples are prepared for each site. The samples are stored above liquid nitrogen below the glass transition temperature of water (below −130 °C), which ensures that biological and chemical changes are reduced to a minimum. In addition, an inert-gas phase in the storage containers created by evaporating nitrogen prevents oxidation processes [46].

### 2.3. Materials and Chemicals

2,4,6-Trinitrotoluene (98.9% purity, 1 mg/mL, in acetonitrile (ACN):methanol (MeOH) 50:50), 1,3-dinitrobenzene (97.0% purity, 1 mg/mL, in ACN:MeOH 50:50), 2,4-dinitrotoluene (98.3% purity, 1 mg/mL, in ACN:MeOH 50:50), 4-amino-2,6-dinitrotoluene (98.4% purity, 1 mg/mL, in ACN:MeOH 50:50), and 2-amino-4,6-dinitrotoluene (97.8% purity, 1 mg/mL, in ACN:MeOH 50:50) were purchased from AccuStandard, New Haven, CT, USA. Isotopically labeled TNT (^13^C_7_, 99%; ^15^N_3_, 98%, 1 mg/mL in benzene, wetted with >33% H_2_O) was purchased from Cambridge Isotope Laboratories, Inc., Andover, MA, USA. Acetonitrile (UHPLC-grade, purity ≥ 99.97%) was purchased from Th. Geyer (Renningen, Germany) and used without further purification. CHROMABOND Easy polystyrene-divinylbenzene-copolymer reversed-phase solid-phase extraction columns (80 µm, 3 mL/200 mg) were purchased from Macherey Nagel, Düren, Germany. Ultrapure water (18.2 MΩ cm) was prepared on-site with a Veolia ELGA Purelab Flex system.

### 2.4. Extraction and GC-MS/MS Analyses

Blue mussel homogenates were retrieved from the German ESB archive and lyophilized at the Fraunhofer Institute for Molecular Biology and Applied Ecology IME (Schmallenberg, Germany). Samples were extracted as described by Bünning et al. [47]. In brief, aliquots of 150–500 mg lyophilized mussel flesh were weighed into lightproof 5 mL tubes. Next, 1.9 mL acetonitrile and 100 µL of a 50 ng/mL ^13^C^15^N-TNT solution were added as internal standard, and samples were vortexed for 60 s, sonicated for 15 min, and centrifuged at 4100 rpm (4 °C) for 15 min. Supernatants were transferred into 25 mL graded flasks, made up to 25 mL with ultrapure water, and introduced onto unconditioned Chromabond Easy SPE columns using a mild vacuum. Columns were then dried i.vac. for 30 min. Samples were eluted with 4 mL ACN, concentrated to 600 µL, and stored at −80 °C in 1.5 mL amber vials [47]. A Thermo Scientific TRACE 1310 gas chromatograph (GC) equipped with a programmable temperature vaporization (PTV) injector and a TriPlus 100 LS autosampler and coupled to a TSQ 8000 EVO triple quadrupole mass spectrometer was used. Large-volume injections (5 µL) were carried out on quartz wool liners (2 mm × 2.75 mm × 120 mm, Thermo Fisher Scientific Inc., Waltham, MA, USA). After injection at 70 °C, a solvent split was performed at 50 mL∗min^−1^ for 0.18 min, followed by a transfer phase of 1.5 min without split flow and a heating rate of 5 °C∗s^−1^ to 240 °C. The injector was finally heated out at 240 °C and a split flow of 200 mL∗min^−1^. The GC was equipped with a TraceGold TG-5MS amine 15 m × 0.25 mm × 0.25 µm column (Thermo Fisher Scientific Inc., Waltham, MA, USA). After an initial phase of one minute at 100 °C, the column was heated at 35 °C∗min^−1^ to 220 °C (held for 0.7 min) and then baked out at 70 °C∗min^−1^ to 280 °C (1 min). Helium served as carrier gas for the GC (1.2 mL∗min^−1^) and argon as collision gas for the mass spectrometer (both Alphagaz, purity of 99.9995%). The mass spectrometer was operated with an electron ionization source (250 °C transfer line, 300 °C source) in selected reaction monitoring mode. Data were recorded and analyzed in Chromeleon 7.2 (Thermo Fisher Scientific Inc., Waltham, MA, USA). Mussel samples were analyzed for the presence of TNT, 1,3-dinitrobenzene (1,3-DNB), 2,4-dinitrotoluene (2,4-DNT), 4-ADNT, and 2-ADNT, and the signal intensity was normalized to the amount of the sample material. Retention times and selected reaction monitoring transitions for these compounds are given in Table 1. Matrix-specific limits of detection (LoDs) and limits of quantification (LoQs) are shown in Table 2. The limit of detection (LoD) and limit of quantification (LoQ) are two important performance characteristics in the validation of a method. Both terms are used to describe the smallest concentration of an analyte that can be reliably measured by an analytical procedure. In more detail, the LoQ stands for the smallest amount or the lowest concentration of a substance that it is possible to determine by means of a given analytical procedure with the established accuracy, precision, and uncertainty. LoD is the lowest analyte concentration likely to be reliably distinguished from the blanks and at which detection is feasible. The LoQ may be calculated based on the standard deviation in the response of the slope of the calibration curve at levels approximating 3.3 times the LoD [48].

## 3. Results

**Figure 2:**
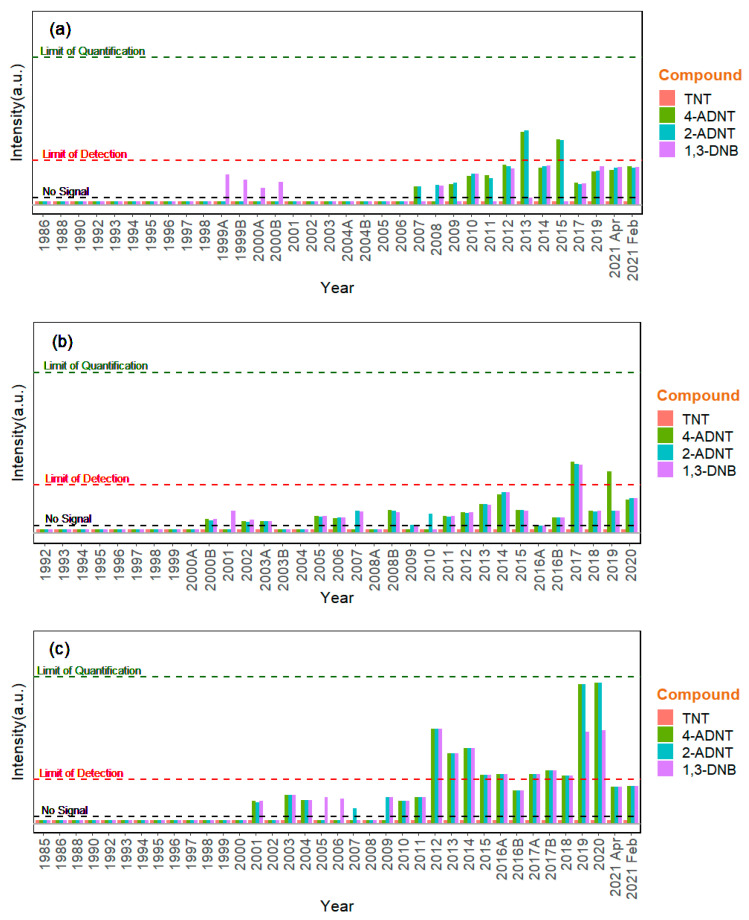
Energetic compounds in blue mussel samples from (**a**) Königshafen (Sylt), (**b**) Darßer Ort, and (**c**) Eckwarderhörne. A and B denote samples received from the same year treated in two different aliquots, which were both analyzed for quality assurance.

Energetic compounds were not detected in the blue mussel samples collected from any of the three sampling regions between the 1980s and end of the 1990s. The first signals indicating the presence of traces of 1,3-DNB were observed in the samples from 1999 and 2000 collected in the North Sea region Königshafen (Sylt) (Figure 2a). The mass spectrometry signals obtained could be assigned to this compound in comparison with spiked samples and blank measurements, although they were still below the calculated LoD (Table 2). Interestingly, in the early 2000s, traces of energetic compounds were also detected (below the LoD) in the blue mussels from the other two ESB sampling regions (Figure 2b,c). For the Baltic Sea region Darßer Ort, this was the case from 2000 onwards (Figure 2b). For the North Sea region Eckwarderhörne, the first signals were observed in blue mussels from 2001, although not consistently in samples from the following years (Figure 2c). At this site, from 2012 onwards, signals of the TNT metabolites 2- and 4-ADNT, as well as 1,3-DNB, were detected in mussels just above the LoD but below the limit of quantification (LOQ) (Figure 2c).

In Königshafen, the other North Sea sampling site (Sylt), and Darßer Ort on the Baltic coastline, the signals for energetic compounds remained below the limits of detection in the subsequent years, with the exception of 2013 and 2015 for Königshafen and 2015, 2017, and 2019 for Darßer Ort, where the limit of detection was just exceeded. The highest signal intensities for 2-ADNT and 4-ADNT, just below the limits of quantification (0.14 ng/g dry weight for 2-ADNT and 0.17 ng/g dry weight for 4-ADNT), were those measured in Eckwarderhörne in 2019 and 2020 (Figure 2c). However, the signal intensities in the most recent mussel samples from 2021 were similar to the respective ranges in samples from 2012 to 2018.

## 4. Discussion

Since the First World War, considerable amounts of munition materials have entered the marine environment worldwide through war actions, such as bombardments or the securing of waterways with mine barriers, or by being dumped at various sites by allies in efforts to demilitarize the defeated nations. Now, 75 years after the end of the Second World War, corrosion of the shells of UXOs and discarded munition materials is expected and energetic compounds will be released into the marine environment. Due to human activities, such as construction of offshore wind farms, submerged munitions need to be removed from these areas, which is usually undertaken by detonating them. Due to incomplete burning of the energetic compounds, pieces of them remain on the seafloor and enter the marine environment as dissolved explosive chemicals. Energetic compounds, such as TNT and its metabolites (2- and 4- ADNT), can be harmful to the marine environment and human health. A large number of studies have shown acute toxic and chronic effects in various marine organisms; human health may also be significantly affected [13,20]. TNT and its metabolites have been shown to cause carcinogenic and mutagenic effects in various organisms due to their ability to cause oxidative stress [17,20,49]. Theoretically, the presence of even one molecule of an energetic compound in an organism can lead to unrecognizable and, ultimately, irreparable cellular damage. It is, therefore, very difficult to set non-harmful limits for energetic compounds, as it is not yet possible to assess the effects of chronic exposure to even low concentrations. Sublethal effects were not expected at these low concentrations in the mussels investigated here. Schuster et al. [19] detected TNT biomarker responses in mussels only at concentrations above 0.31 mg/L, much higher than the concentrations in water expected for the monitored sites.

The parent substance TNT was not detected in any of the mussel samples obtained from the German ESB. However, its metabolites 2- and 4-ADNT were present in the animals, which may have been formed by mussel-specific metabolic enzymes, by microorganisms living in the soft body or on the mussel shells, or in the surrounding sea water [27,34]. In recent field experiments, 2- and 4-ADNT were the predominant metabolites found in exposed blue mussels, with 4-ADNT in significantly higher amounts than 2-ADNT [18,28,29]. TNT was only found in mussels that were in close proximity to explosives, such as exposed hexanite (without metal shells) [18,29]. Laboratory experiments have confirmed that 2-ADNT—and, especially, 4-ADNT—are the dominant energetic compounds in mussels; TNT itself was only measured to a minor extent compared to its metabolites [15,19]. The metabolites 2- and 4-ADNT were also detected in higher concentrations in the bile of juvenile Atlantic salmon (*Salmo salar*) exposed to TNT than TNT itself in a laboratory study [50]. Ek et al. [51] even found up to 100-fold higher concentrations of 2- and 4-ADNT than TNT in the bile of rainbow trout (*Oncorhynchus mykiss*) exposed to TNT in the laboratory. Bile from randomly caught dab (*Limanda limanda* L., from the Baltic Sea) contained 4-ADNT in significantly higher concentrations than 2-ADNT and TNT [24]. Recently, TNT and its metabolites 2- and 4-ADNT, as well as 1,3-DNB, have been found in fillets of pouting (*Trisopterus luscus*) living at the wreck site of a sunken Second World War ship in the North Sea [7].

In recent years, a number of studies have shown that the metal casings of UXOs and dumped munitions exposed to seawater corrode over time (e.g., [9,10]). Several studies have already shown that energetic compounds appear in the water column of the North and Baltic Sea adjacent to point sources, such as dumping sites [1,25] or ship wrecks [7], and were detectable in blue mussels (*Mytilus* spp.) exposed to corroding submerged munitions and uncovered explosive materials [18,28,29]. Greinert et al. [52] have recently provided a regional-scale distribution of dissolved TNT in bottom waters throughout the southwest of the German Baltic Sea. They have shown TNT concentrations ranging between 0 pM and 354 pM. The differences observed were suggested to be due to the distance from munition dumpsites, the water currents, and the deepness of the water column. Nevertheless, munition chemicals are widespread in the Baltic Sea and make it challenging to link specific TNT sources to the presence of TNT in any biota. Although the conclusion that increasing corrosion can be expected to result in increasing concentrations of energetic compounds in the water column should not be disputed, a study by den Otter et al. [11] reported unexpectedly low water column contamination despite the advanced state of corrosion of the underwater munitions at their study site.

Analysis of blue mussels from the German Environmental Specimen Bank was initiated to provide further insights into the occurrence of energetic compounds in biota from major battleground areas of the First and Second World Wars. This can not only facilitate the monitoring of potential hazards from munition residues for human health and ecosystems in areas that have been exposed to munitions via different routes during and after the World Wars, but also provides much needed environmental data on the progression of the corrosion of dumped munitions in the German coastal regions and the extent to which energetic compounds leached from these munitions are taken up by marine organisms.

As mussel sampling for ESB is always conducted at randomly selected sites in sampling areas of a few square kilometers, the frequent detections in recent years are probably not due to individual munitions remnants but rather indicate more diffuse contamination of sampling areas with munitions relics. These may result from dumped munitions or ship wrecks in the sampling region or from clearance operations and deliberate detonations, such as blast-in-place operations conducted to destroy munitions that are not safe to handle or too far offshore to bring ashore. Where possible, these operations are carried out in shallow water or on sandbanks to avoid impacting the hearing of marine mammals, such as harbor porpoises. It is known that low-order detonations under water, in particular, lead to incomplete combustion of the explosives [18,25,29]. As a consequence, the energetic compounds can spread from the site of the explosion over a large area in small particles that are more water-soluble in the sea water than larger fragments [18,25,29]. Therefore, higher concentrations of energetic compounds in water are expected where these remediation activities occur, eventually resulting in the uptake of these compounds in marine biota.

Submerged munitions are known to be present in all three regions investigated in this study. On the one hand, discarded munition materials are located in defined dumping areas, such as near the German ESB sampling site Eckwarderhörne. However, it cannot be ruled out that dumped munitions might also be located outside these defined areas, as navigation after the Second World War was not as precise as in modern times. Furthermore, so-called “on-route-dumping”, the deliberate release of munitions before reaching the designated dumping areas, was apparently a common practice in the post-war years [4,53]. For instance, considerable amounts of munitions are suspected to have been dumped unrecorded between the island of Sylt and the German mainland from fortifications that were built on the island during the Second World War. In addition, there were several air defense units and formations on the island directed at the surrounding Wadden Sea, which likely added to the munition relics. In recent years, there have been repeated findings of munitions in the vicinity of the German ESB sampling area near Königshafen, Sylt, as reported by Böttcher et al. [54]. The ESB sampling area is located in a small bay where aerial bombs and small munitions have also been found [55]. In addition, several wrecked combat aircraft are located directly at the entrance of the bay and sea mines have also been found in the sampling site in past years [55]. The German ESB data reported in this study indicate that blue mussels from Sylt island are increasingly exposed to explosive chemicals released into the environment as a result of the progressive corrosion of the munition shells in this area.

The sampling area Darßer Ort in the Bodden National Park of Western Pomerania has been affected by military action in the Second World War, munition dumping during and after the war, and maneuvers conducted by the former German Democratic Republic (GDR). In general, data on munition dumping are much less accessible for this region in the GDR, which was under the military administration of the USSR, than for the North Sea, a British occupation zone in the Federal Republic of Germany. However, the ESB sampling region covers a known munitions dumping ground, and reports confirm that munitions have been dumped in the immediate vicinity of the German ESB sampling site [55]. Another potential source of munitions are missiles and up to 30 mm caliber ammunition shot at sea targets during military training for the GDR navy [55]. There are no reports on the presence of munitions in the ESB region during the years of sampling [55], while military training in the GDR stopped in the late 1980s and all Second World War-related exposure dates back to the 1940s and 1950s. It can, therefore, be suspected that any contamination of the mussels with energetic compounds originates entirely from munition relics in the sampling region, which started to release explosives after corrosion.

**Figure 3:**
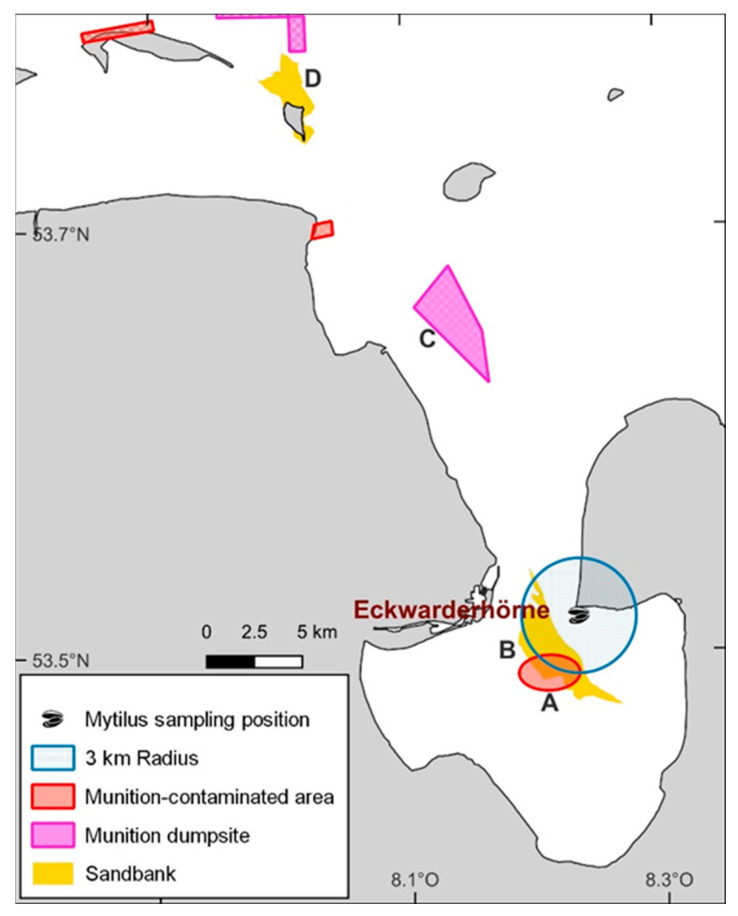
Map of the *Mytilus* sampling area of the Environmental Specimen Bank “Eckwarderhörne” in the German North Sea. (A) The munition-contaminated area Jappensand, (B) Jappensad shoal, (C) the munition dumpsite Hooksielplate, (D) the island Minsener Oog with shoal.

The sampling area Eckwarderhörne is located directly at the entrance of the Jade Bight. It is estimated that up to 1.2 million tons of conventional munitions are located in this area, most of them in the munition dumping ground Hooksielplate (Figure 3) [4,56]. This area is also expected to experience an increasing release of energetic compounds due to the ongoing corrosion of munitions shells. The ongoing corrosion also explains the increasing presence of energetic compounds in the mussels from Eckwarderhörne analyzed in this study. For instance, bombs and smoke bombs were recovered in the immediate vicinity of the sampling area between 2006 and 2007 [55]. More frequent detections of TNT metabolites in the mussels from 2012 onwards could also be explained by, for example, targeted blasting of shells and bombs on the Jappensand shoal, an area in the immediate vicinity of the sampling region (Figure 3) [55]. For instance, 104 type MK11/MK6 water bombs were found and target blasted on the northern part of the Jappensand shoal in April 2011 [4]. Munitions blasting on other sandbanks in the vicinity of Jade Bight could also result in short-term increases in the concentration of energetic compounds in the German ESB mussels as contaminated water is pushed into Jade Bay with the onset of high tide. For example, UXOs have now been deliberately detonated on Minsener Oog for several years (personal communication with responsible parties in 2021), such as in 2014, when 24 moored mines were target blasted on Minsener Oog [57].

This study clearly shows that corroding submerged munitions have started to gradually release energetic compounds into the marine environment and that these toxic and carcinogenic chemicals could be detected in randomly sampled blue mussels at three sites on the German North and Baltic Sea coast. In addition, explosive residues from blast-in-place operations may have added to the exposure in the North Sea ESB sampling sites. Overall, it appears that the release of energetic compounds started in the beginning of the 21st century, 55 years after the end of the Second World War. Even though the concentrations measured are still in the non-quantifiable trace range, it can be concluded that the levels and abundances of these energetic compounds are increasing. It cannot, therefore, be ruled out that this problem could worsen in the coming years if the munitions are not removed from the seas. TNT leakage from dumped conventional munitions may thus add to a cocktail of anthropogenic pollutants, such as microplastics, pesticides, and pharmaceuticals, which is threatening the marine environment. In addition, TNT and its metabolites are classified as possible mutagenic and carcinogenic chemicals, such that a risk for the human seafood consumer upon intake of energetic compounds by fish, mussels, and shrimps cannot be ruled out.

## 5. Conclusions

Generally, the development of monitoring and detection methods is of high relevance for the enhancement of the localization and mapping of munitions on the seafloor, especially since the exact locations of large amounts of munitions remain unknown. In addition to active biomonitoring using blue mussels (*Mytilus* spp.) and passive sampling systems, such as POCIS, sediment and water samples may routinely be analyzed. This study illustrates the usefulness of long-term archiving of environmental samples: while recent advances in analytical instrumentation provide suitable methods for the analysis of energetic compounds in environmental samples, these techniques were not available in the past. In the future, continuous monitoring of German ESB sites for energetic compounds together with analysis of archived mussels from other ESBs can add a much-needed historical perspective to current investigations and thus critically improve environmental management and risk communication.

Retrospective analysis of ESB mussel time series showed that environmental contamination from TNT-based munitions is increasing along the sampling areas in the North and Baltic Sea. The data necessitate further monitoring in coastal regions that were battlegrounds in both World Wars and dumping grounds for Second World War munitions. The potential main source is leaking energetic compounds after progressive corrosion of metal casings of dumped munitions over the last decades. More recent exposure may originate from targeted blasting of submerged munitions in the sea or on sandbanks, which should be avoided because they temporarily release considerable quantities of explosive compounds into the environment and due to the underwater noise harming marine mammals. Relic munitions still lying in the sea should be recovered as soon as possible and safely destroyed on land or on a floating platform, as progressive corrosion of metal casings will hinder their identification in the future. The retrieval of submerged munitions should, therefore, be integrated into the EU Zero Pollution Ambition and the goals of the Marine Strategy Framework Directive in order to remove these legacy contaminations from European seas and avoid long-lasting damage to ecosystem and human health.

## Figures and Tables

**Table 1:** GC retention times and MS/MS transitions for the energetic compounds examined. Adapted from Bünning et al. 2021 [47].

Compound		Rt SL (min)	Rt LVI (min)	Molecular Mass (g∗mol^−1^)	Transition (m/z)		CE (eV)
1,3-Dinitrobenzene CAS No. 99-65-0	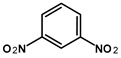	2.43	3.20	168.11	Q q q	122.0 > 75.0 168.0 > 75.0 168.0 > 122.0	12 20 8
2,4-Dinitrotoluene CAS No. 121-14-2	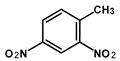	2.77	3.52	182.13	Q q q	165.0 > 63.1 165.0 > 90.1 165.0 > 118.1	22 16 8
Trinitrotoluene CAS No. 118-96-7	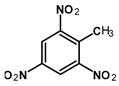	3.41	4.09	227.13	Q q q	210.0 > 164.1 164.0 > 90.1 108.0 > 76.1	6 10 12
^13^C^15^N-Trinitrotoluene CAS No. 202406-62-0	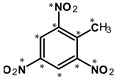	3.41	4.09	237.06	Q q q	220.1 > 173.1 220.1 > 203.1 189.1 > 82.1	6 8 10
4-Amino-2,6-dinitrotoluene CAS No. 19406-51-0	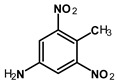	4.22	4.85	197.15	Q q q	197.0 > 180.1 180.0 > 163.1 163.0 > 78.0	6 8 14
2-Amino-4,6-dinitrotoluene CAS No. 35572-78-2	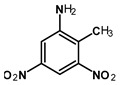	4.42	5.07	197.15	Q q q	197.0 > 180.1 180.0 > 133.0 180.0 > 67.0	6 6 12

Abbreviations: Rt SL = retention time split/splitless; Rt LVI = retention time large-volume injector; Q = quantifying ion; q = qualifying ion; CE = collision energy. All carbon and nitrogen atoms marked with asterisks were isotopically pure ^13^C or ^15^N.

**Table 2:** Limits of detection (LODs) and quantification (LOQs) of the method used [47].

Compound	LOD (ng/g d.w.)	LOQ (ng/g d.w.)	R^2^
1,3-Dinitrobenzene	0.03	0.10	0.985
2,4-Dinitrotoluene	0.04	0.12	0.990
2,4,6-Trinitrotoluene	0.20	0.68	0.986
4-Amino-2,6-dinitrotoluene	0.05	0.17	0.973
2-Amino-4,6-dinitrotoluene	0.04	0.14	0.980

## Data Availability

Data are contained within the article.
